# Mitogenome data of *Nycticebus coucang insularis* Robinson, 1917 (Primate: Lorisidae)

**DOI:** 10.1016/j.dib.2019.104058

**Published:** 2019-05-25

**Authors:** Jeffrine J. Rovie-Ryan, Millawati Gani, Yin Peng Lee, Han Ming Gan, Mohd Tajuddin Abdullah

**Affiliations:** a National Wildlife Forensic Laboratory (NWFL), Ex-Situ Conservation Division, Department of Wildlife and National Parks (PERHILITAN) Peninsular Malaysia, KM 10 Jalan Cheras, 56100 Kuala Lumpur, Malaysia; b Institute of Tropical Biodiversity and Sustainable Development, Universiti Malaysia Terengganu, 21030 Kuala Nerus, Terengganu, Malaysia; c Monash University Malaysia Genomics Facility, Tropical Medicine and Biology Multidisciplinary Platform, 47500 Bandar Sunway, Selangor Darul Ehsan, Malaysia; d School of Life & Environmental Sciences, Deakin University, Geelong, Victoria 3220 Australia; e Deakin Genomics Centre, Deakin University, Geelong, Victoria 3220 Australia

**Keywords:** *Nycticebus coucang insularis*, Mitogenome, Tioman island

## Abstract

This data article presents the first complete mitochondrial genome (mitogenome) of an endangered slow loris subspecies, *Nycticebus coucang insularis* Robinson, 1917 from Tioman Island, Pahang. Once considered as extinct, an individual of the subspecies was captured alive from the island during the 2016 Biodiversity Inventory Programme as highlighted in the related research article entitled “Rediscovery of *Nycticebus coucang insularis* Robinson, 1917 (Primates: Lorisidae) at Tioman Island and its mitochondrial genetic assessment” Rovie-Ryan et al., 2018. Using MiSeq™ sequencing system, the entire mitogenome recovered is 16,765 bp in length, made up of 13 protein-coding genes, two rRNA genes, 22 tRNA genes, and one control region. The mitogenome has been deposited at DDBJ/EMBL/GenBank under the accession number NC_040292.1/MG515246.

**Table:** Specifications table

Subject area	Genomics
More specific subject area	Mitogenomics
Type of data	Morphological measurements, tables, mitogenome sequence data in FASTA file format, photographs in JPEG image file format, figures in PNG image file format
How data was acquired	Buccal swab DNA sampling, DNA was extracted using QIAamp^®^ DNA Mini Kit (Qiagen, Germany), Hardware used for analysis includes M220 Focused-ultrasonicator (Covaris, USA) and MiSeq™ Benchtop Sequencer (Illumina, USA), NEBNext Ultra DNA Library Prep Kit for Illumina (New England Biolabs, Ipswich, MA) was used for sequencing, Softwares for analyses includes BOWTIE2, GENEIOUS v10.1.3, MITOS annotation web service, and MEGA v7
Data format	Raw, semi-analyzed, and analyzed
Experimental factors	Assembly of short read sequences to construct complete mitogenome sequence, phylogenetic analysis, bootstrap test
Experimental features	Genomic DNA was extracted from the buccal swab sample. The complete mitogenome was sequenced and assembled by using BOWTIE2 as a plugin in GENEIOUS v10.1.3. Phylomitogenomics relationship was constructed using MEGA v7
Data source location	The individual was caught at Kampung Sungai Asah, Tioman Island, State of Pahang, Malaysia (Latitude: 2°43′16.32″N Longitude: 104°11′40.93″E)
Data accessibility	Voucher specimen of the specimen is kept at the Wildlife Genetic Resource Bank (WGRB) of PERHILITAN (voucher number = NC37). The mitogenome data is available at DDBJ/ENA/GenBank under the accession number NC_040292/MG515246. https://www.ncbi.nlm.nih.gov/nuccore/MG515246.1https://www.ncbi.nlm.nih.gov/nuccore/NC_040292.1
Related research article	Rovie-Ryan, J.J., Gani, M., Gan, H.M., Bolongon, G.G., Cheng, T.C., Razak, N., Rosli, N., Aziz, M.A. & Matkasim, K. (2018). Rediscovery of *Nycticebus coucang insularis* Robinson, 1917 (Primates: Lorisidae) at Tioman Island and its Mitochondrial Genetic Assessment. *Sains Malaysiana*, *47* (10), 2533–2540 [1].

**Table:** 

**Value of the data** • This data reported here is the first mitogenome of Nycticebus coucang insularis Robinson, 1917 and the only mitogenome data available from two specimens ever collected from this subspecies • The data can benefit primatologist, molecular ecologists, and geneticists working on evolutionary and phylogeography studies • The current data can be used to elucidate the phylomitogenomics of the genus Nycticebus • This data provide the essential reference for the management authorities for genetic management pool of Nycticebus metapopulations in Southeast Asia

## 1 Data

**Fig. 1:**
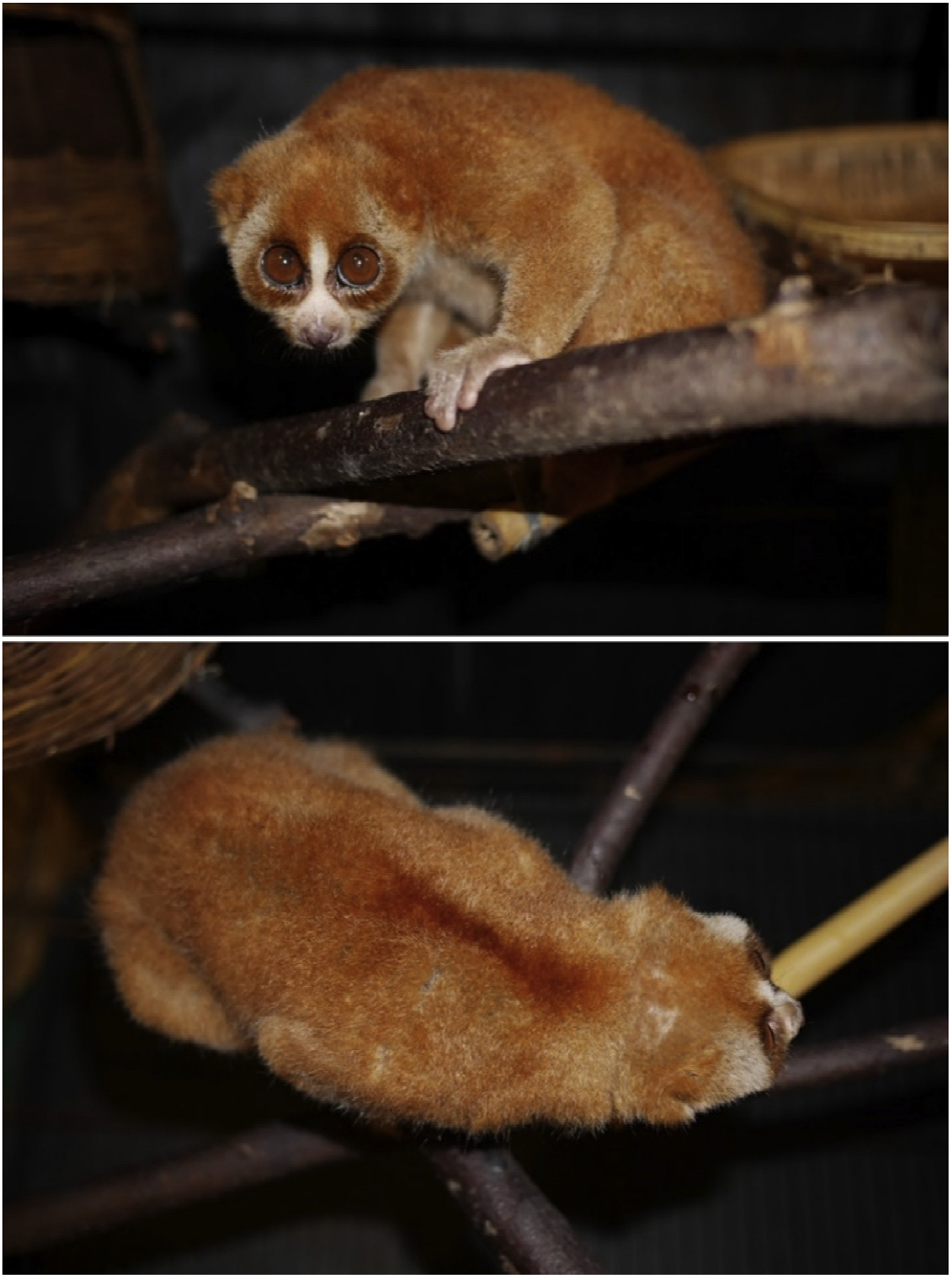
Photographs of the individual (*Nycticebus coucang insularis*) caught at Kampung Sg. Asah, Tioman Island during the recent 2016 Biodiversity Inventory Programme (photographed by Cheng T.C.).

**Fig. 2:**
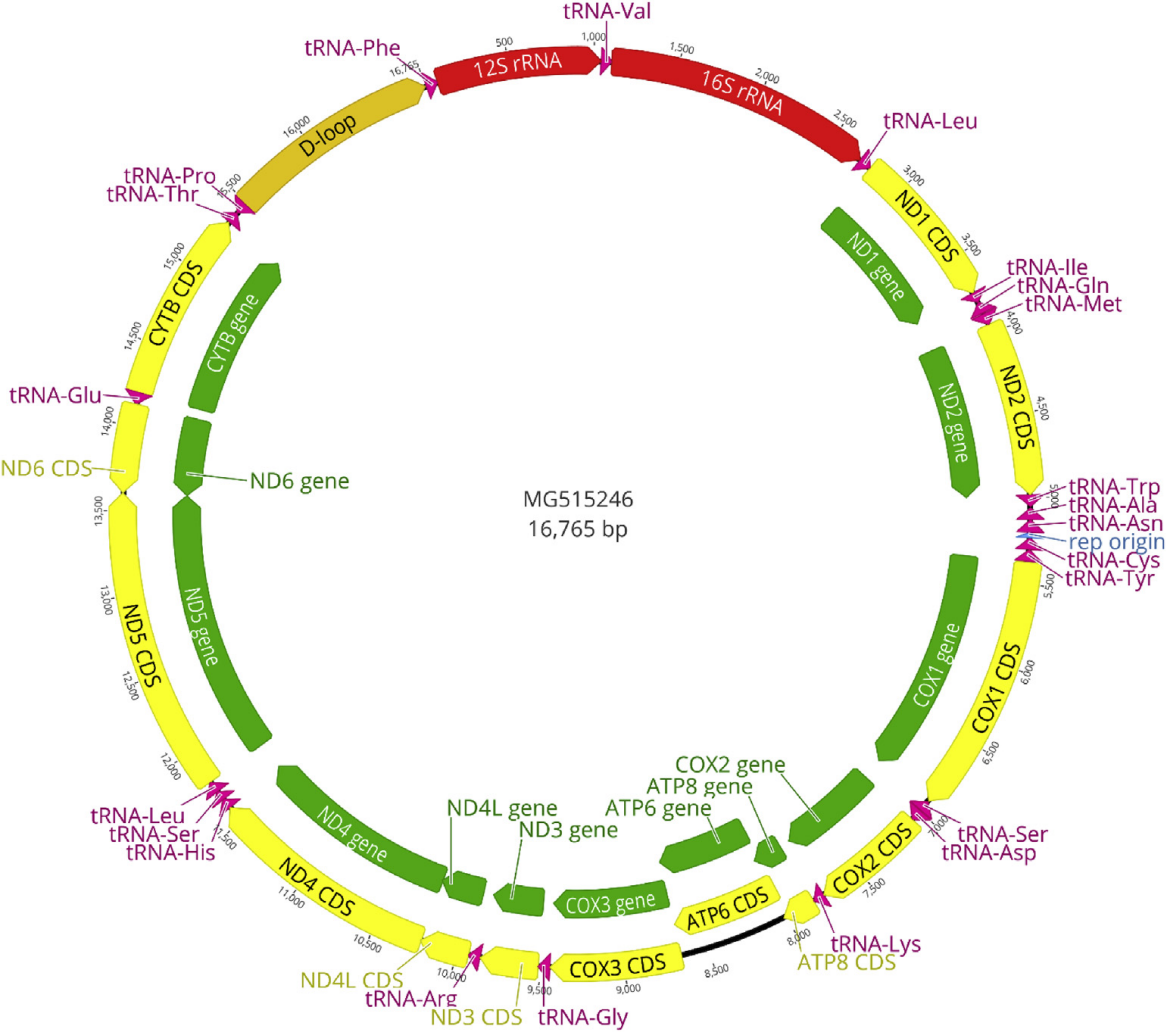
The complete mitogenome of *N. coucang insularis*.

**Fig. 3:**
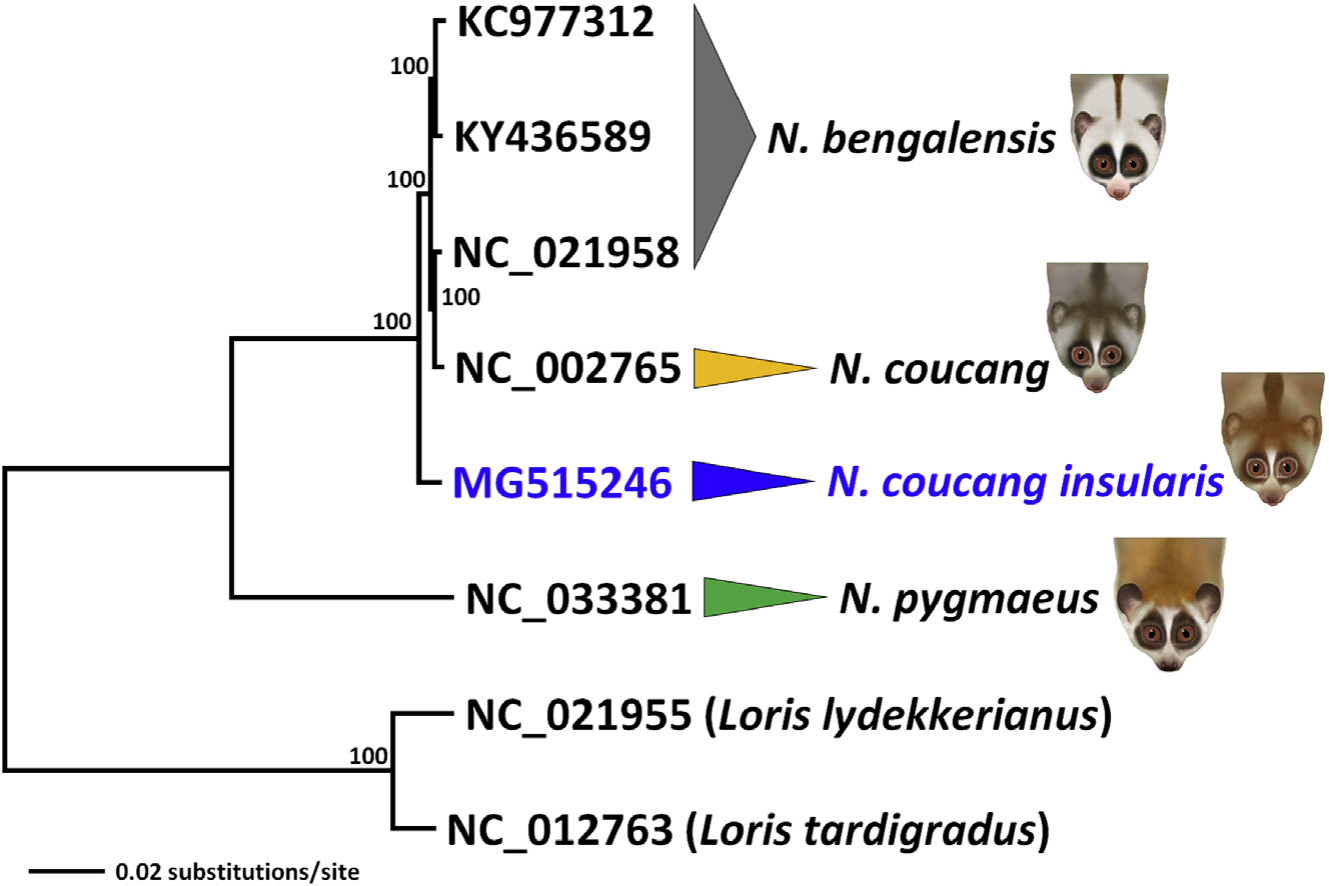
Phylomitogenomic relationship of the genus *Nycticebus* as constructed using the neighbor-joining method. Illustrations of *Nycticebus* were taken from [9] with permission.

*Nycticebus coucang insularis* Robinson, 1917 was speculated to have extinct in Tioman Island (State of Pahang, Malaysia) [2] until the rediscovery of an individual during the 2016 Biodiversity Inventory Programme [1]. Morphological measurements of the individual (total length = 279 mm, head body = 263 mm, tail = 16 mm, ear = 18 mm, hind foot = 45 mm and weight = 500 g) and photographs were taken (Fig. 1). Here, we present the mitogenome of the specimen which was caught at Kampung Sungai (Sg.) Asah, Tioman Island (Latitude: 2°43′16.32″ Longitude: 104°11′40.93″). We provided a table (Table 1) and figure (Fig. 2) of the gene organization of the mitogenome and calculated the genetic distances among the *Nycticebus* mitogenomes (Table 2). We also provided the phylomitogenomic tree construction of among the available mitogenomes of *Nycticebus* (Fig. 3).

**Table 1:** The mitochondrial genome organization of N. coucang insularis.

Gene	Start	End	Orientation	Length (bp)
tRNA-Phe	1	71	forward	71
12S rRNA	72	1,042	forward	971
tRNA-Val	1,043	1,110	forward	68
16S rRNA	1111	2699	forward	1,589
tRNA-Leu	2,700	2,775	forward	76
ND1 gene	2776	3730	forward	955
tRNA-Ile	3,731	3,799	forward	69
tRNA-Gln	3,797	3,868	reverse	72
tRNA-Met	3,872	3,940	forward	69
ND2 gene	3941	4982	forward	1,042
tRNA-Trp	4,983	5,049	forward	67
tRNA-Ala	5,059	5,126	reverse	68
tRNA-Asn	5,128	5,200	reverse	73
rep origin	5,201	5,233	reverse	33
tRNA-Cys	5,234	5,300	reverse	67
tRNA-Tyr	5,301	5,367	reverse	67
COX1 gene	5,380	6,921	forward	1,542
tRNA-Ser	6,923	6,990	reverse	68
tRNA-Asp	6,996	7,064	forward	69
COX2 gene	7,065	7,748	forward	684
tRNA-Lys	7,751	7,819	forward	69
ATP8 gene	7,820	8,023	forward	204
ATP6 gene	7,981	8,661	forward	681
COX3 gene	8,661	9,444	forward	784
tRNA-Gly	9,445	9,513	forward	69
ND3 gene	9,514	9,860	forward	347
tRNA-Arg	9,861	9,929	forward	69
ND4L gene	9,930	10,226	forward	297
ND4 gene	10,220	11,603	forward	1,384
tRNA-His	11,604	11,666	forward	63
tRNA-Ser	11,667	11,724	forward	58
tRNA-Leu	11,725	11,794	forward	70
ND5 gene	11,795	13,606	forward	1,812
ND6 gene	13,603	14,130	reverse	528
tRNA-Glu	14,131	14,199	reverse	69
CYTB gene	14,203	15,342	forward	1,140
tRNA-Thr	15,347	15,414	forward	68
tRNA-Pro	15,417	15,485	reverse	69
D-loop	15,486	16,765	forward	1,280

**Table 2:** Genetic distances (in percentage, %) calculated among the Nycticebus mitogenomes using the Kimura 2-parameter model as implemented in MEGA v7.

	Species/Subspecies	1	2	3	4
1.	*N. coucang insularis*				
2.	*N. coucang*	1.145			
3.	*N. bengalensis*	1.133	0.504		
4.	*N. pygmaeus*	11.387	11.402	11.387

## 2 Experimental design, materials, and methods

Genomic DNA (gDNA) was extracted from the buccal swab sample using QIAamp^®^ DNA Mini Kit (Qiagen, Germany). gDNA was later sheared using the M220 Focused-ultrasonicator (Covaris, USA) and the library was prepared using NEBNext Ultra DNA Library Prep Kit for Illumina (New England Biolabs, Ipswich, MA) according to the manufacturer's protocol and sequenced on the MiSeq™ Benchtop Sequencer (2 × 250 bp paired-end reads) (Illumina, USA). BOWTIE2 [3] as a plugin in GENEIOUS v10.1.3 [4] was used to assemble a total of 1,318,109 short read sequences to construct reliable mitogenome using a reference sequence of *Nycticebus coucang* (GenBank Accession number: NC_002765). The mitogenome was annotated using the MITOS annotation web service [5]. In summary, the entire mitogenome is 16,765 bp in length which are made up of 13 protein-coding genes, two rRNA genes, 22 tRNA genes, and one control region as summarized in Table 1 while Fig. 2 showed the gene organization. Genetic distances among *Nycticebus* were also calculated using the Kimura 2-parameter model [6] implemented in MEGA v7 [7] as shown in Table 2.

To construct the phylomitogenomic relationship within the genus *Nycticebus*, available mitogenomes of *N. bengalensis* (KC977312, KY436589, and NC_021958), *N. coucang* (NC_002765), and *N. pygmaeus* (NC_033381) were aligned using MUSCLE [8] as implemented in GENEIOUS v10.1.3. The neighbor-joining method was used for the phylomitogenomic tree construction (Fig. 3) as implemented in MEGA v7 with 2000 bootstrap replications and Kimura 2-parameter model.
